# 58. Cost-Effectiveness of Emerging Antibiotic Strategies for the Treatment of Drug-Use Associated Infective Endocarditis

**DOI:** 10.1093/ofid/ofab466.058

**Published:** 2021-12-04

**Authors:** Joella W Adams, Alexandra Savinkina, Mam Jarra Gai, Allison Hill, James Hudspeth, Raagini Jawa, Simeon D Kimmel, Laura Marks, Benjamin P Linas, Joshua Barocas

**Affiliations:** 1 RTI, Barrington, RI; 2 Boston Medical Center (BMC), Boston, Massachusetts; 3 Boston Medical Center, Boston, Massachusetts; 4 Boston University School of Medicine and Boston Medical Center, Boston, Massachusetts; 5 Washington University in St. Louis, St. Louis, MO; 6 Boston University School of Medicine/Boston Medical Center, Boston, MA; 7 University of Colorado Anschutz Medical Campus, Aurora, Colorado

## Abstract

**Background:**

Drug use-associated infective endocarditis (DUA-IE) is typically treated with 4-6 weeks of in hospital intravenous antibiotics (IVA). Outpatient parenteral antimicrobial therapy (OPAT) and partial oral antibiotics (PO) may be as effective as IVA, though long-term outcomes and costs remain unknown. We evaluated the clinical outcomes and cost-effectiveness of four antibiotic treatment strategies for DUA-IE.

**Methods:**

We used a validated microsimulation model to compare: 1) 4-6 weeks of inpatient IVA along with opioid detoxification, *status quo* (SQ); 2) 4-6 weeks of inpatient IVA along with inpatient addiction care services (ACS) which offers medications for opioid use disorder (*SQ with ACS*); 3) 3 weeks of inpatient IVA with ACS followed by OPAT (*OPAT*); and 4) 3 weeks of IVA with ACS followed by PO antibiotics (*PO*). We derived model inputs from clinical trials and observational cohorts. All patients were eligible for either in-home or post-acute care OPAT. Outcomes included life years (LYs), discounted costs, incremental cost-effectiveness ratios (ICERs), proportion of DUA-IE cured, and mortality attributable to DUA-IE. Costs (&US) were annually discounted at 3%. We performed probabilistic sensitivity analyses (PSA) to address uncertainty.

**Results:**

The *SQ* scenario resulted in 18.64 LY at a cost of &416,800/person with 77.4% hospitalized DUA-IE patients cured and 5% of deaths in the population were attributable to DUA-IE. Life expectancy was extended by each strategy: 0.017y in *SQ with ACS*, 0.011 in *OPAT*, and 0.024 in *PO*. The *PO* strategy provided the highest cure rate (80.2%), compared to 77.9% in *SQ with ACS* and 78.5% in *OPAT and X in SQ*. *OPAT* was the least expensive strategy at &412,300/person, Compared to OPAT, *PO* had an ICER of &141,500/LY. Both *SQ* strategies provided worse clinical outcomes for money invested than either OPAT or PO (dominated). All scenarios decreased deaths attributable to DUA-IE compared to *SQ*. Findings were robust in PSA.

Table 1

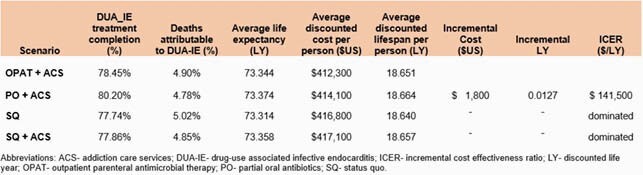

Selected cost and clinical outcomes comparing treatment strategies for drug-use associated infective endocarditis including the status quo, status quo with addiction care services, outpatient parenteral antimicrobial therapy, and partial oral antibiotics.

**Conclusion:**

Treating DUA-IE with OPAT along with ACS increases the number of people completing treatment, decreases DUA-IE mortality, and is cost-saving compared to the status quo. The PO strategy also improves clinical outcomes, but may not be cost-effective at the willingness-to-pay threshold of &100,000.

**Disclosures:**

**Simeon D. Kimmel, MD, MA**, **Abt Associates for a Massachusetts Department of Public Health project to improve access to medications for opioid use disorder in nursing facilities** (Consultant)

